# Supplementary material for: Traditional and Complementary Medicine Use among Cancer Patients in Asian Countries: A Systematic Review and Meta-Analysis
Source: Cancers (Basel). 2024 Sep 11;16(18):3130. doi: 10.3390/cancers16183130 (PMC11429845; doi:10.3390/cancers16183130)
Supplement: Supplementary file 1 [file cancers-16-03130-s001.zip › cancers-3137842-supplementary.pdf]

Supplementary Material

Supplementary Table S1. Search strategies

Search	Query
**1. PubMed/MEDLINE**	
1	((neoplasms[MeSH Terms]) OR (cancer* OR neoplasm* OR malignan* OR carcinoma* OR oncolog* OR eukemi* OR lymphoma*))
2	((complementary therapies [MeSH Terms]) OR (herbal medicine [MeSH Terms]) OR (complementary medic*) OR (complementary therap*) OR (alternative medic*) OR (alternative therap*) OR (integrative medic*) OR (integrative therap*) OR (traditional medic*) OR (unconventional medic*) OR (unconventional therap*) OR (Non-hospital treat*) OR (herbal medic*) OR (herbal therap*))
3	1 AND 2
4	((Surveys and Questionnaires [MeSH Terms]) OR ("cross sectional studies"[MeSH Terms]) OR (survey) OR ("cross-sectional study") OR ("cross-sectional survey"))
5	3 AND 4
6	(names of Asian countries[†] using OR in between)
7	5 AND 6
8	("meta-analysis"[Title] OR "meta-analysis"[Publication Type] OR "review"[Title] OR "review"[Publication Type] OR "clinical trial"[Publication Type] OR "randomized controlled trial"[Publication Type] OR "Comment"[Publication Type] OR "Editorial"[Publication Type])
9	7 NOT 8
10	limit 9 to (human, and full text)
**2. Ovid MEDLINE(R) and Epub Ahead of Print, In-Process & Other Non-Indexed Citations, Daily and Versions(R)**	
1	exp complementary medicine/
2	((complementary OR alternative OR integrat* OR traditional OR unconventional OR herbal) adj1 (therap* OR medicine)).tw,kw.
3	1 OR 2
4	exp neoplasms/
5	(cancer* OR neoplasm* OR malignan* OR carcinoma* OR oncolog* OR eukemi* OR lymphoma*)

Search	Query
6	4 OR 5
7	3 AND 6
8	(survey OR questionnaire OR cross-sectional survey OR cross-sectional stud*).af.
9	7 AND 8
10	(names of Asian countries† using OR in between).af.
11	9 AND 10
**3. CINAHL (EBSCO)**	
1	MH "neoplasms+"
2	cancer* OR neoplasm* OR malignan* OR carcinoma* OR oncolog* OR eukemi* OR lymphoma*
3	1 OR 2
4	TI ("Complementary and alternative medicine" OR "Complementary & alternative medicine" OR CAM) OR AB("Complementary and alternative medicine" OR "Complementary & alternative medicine" OR CAM) OR MH "Alternative Therapies+"
5	"complementary therap*" OR "herbal medic*" OR "complementary medic*" OR "alternative medic*" OR "integrative medic*" OR "integrative therap*" OR "traditional medic*" OR "unconventional medic*" OR "unconventional therap*" OR "Non-hospital treat* " OR "herb* medic*" OR "herb* therap*" OR "herb*"
6	4 OR 5
7	3 AND 6
8	TI("cross sectional" OR "cross-sectional") OR AB("cross sectional" OR "cross-sectional") OR MH "Questionnaires" MH "Surveys" OR questionnaire* OR survey*
9	7 AND 8
10	(names of Asian countries† using OR in between)
11	9 AND 10
**4. Web of Science**	
1	cancer* OR neoplasm* OR malignan* OR carcinoma* OR oncolog* OR eukemi* OR lymphoma* (Topic)
2	((complementary therap*) OR (herbal medicine*) OR (complementary medic*) OR (complementary therap*) OR (alternative medic*) OR (alternative therap*) OR (integrative medic*) OR (integrative therap*) OR (traditional medic*) OR

Search	Query
	(unconventional medic*) OR (unconventional therap*) OR (Non-hospital treat*) OR (herbal medic*) OR (herbal therap*)) (All Fields)
3	1 AND 2
4	("cross sectional" OR "cross-sectional") (Title) OR ("cross sectional" OR "cross-sectional") (Abstract) OR ("Questionnaires" OR "Surveys" OR questionnaire* OR survey*) (All Fields)
5	3 AND 4
6	("meta-analysis" OR “meta analysis” OR "review" OR "clinical trial" OR "randomized controlled trial" OR "RCT*"OR "Comment" OR "Editorial") (All Fields)
7	5 NOT 6
8	TI("cross sectional" OR "cross-sectional") OR AB("cross sectional" OR "cross-sectional") OR MH "Questionnaires" MH "Surveys" OR questionnaire* OR survey*
9	7 AND 8
† The list of Asian countries follows the "World Economic Situation and Prospects 2020"s [1] classification criteria: Afghanistan, Bahrain, Bangladesh, Bhutan, Brunei Darussalam, Cambodia, China, Fiji, Hong Kong, India, Indonesia, Iran, Iraq, Israel, Japan, Jordan, Kiribati, Korea*, Lao*, Lebanon, Malaysia, Maldives, Mongolia, Myanmar, Nepal, Oman, Pakistan, Palestine, Papua New Guinea, Philippines, Samoa, Saudi Arabia, Singapore, Solomon Islands, Sri Lanka, Syrian, Taiwan, Thailand, Timor-Leste, Turkey, Vanuatu, Vietnam, Yemen	

[1] United Nations. World Economic Situation and Prospects 2020. UN Department of Economic and Social Affairs. Economic Analysis. 16th Jan. 2020. https://www.un.ORg/development/desa/dpad/publication/wORld-economic-situation-and-prospects-2020/ (last accessed 10th Sep. 2020).

Results of Quality assessment with the Appraisal tool for Cross-Sectional Studies (AXIS tool)

Supplementary Table S2. The proportion to meet individual AXIS criteria

Item	AXIS criteria	meet the criteria (%)
	**Introduction**	
1	Were the aims/objectives of the study clear?	97.6
	**Methods**	
2	Was the study design appropriate for the stated aim(s)?	100.0
3	Was the sample size justified?	19.5
4	Was the target/reference population clearly defined? (Is it clear who the research was about?)	97.6
5	Was the sample frame taken from an appropriate population base so that it closely represented the target/reference population under investigation?	95.1
6	Was the selection process likely to select subjects/participants that were representative of the target/reference population under investigation?	90.2
7	Were measures undertaken to address and categorise non-responders?	2.4
8	Were the risk factor and outcome variables measured appropriate to the aims of the study?	97.6
9	Were the risk factor and outcome variables measured correctly using instruments/measurements that had been trialed, piloted or published previously?	68.3
10	Is it clear what was used to determine statistical significance and/or precision estimates? (e.g. p-values, confidence intervals)	75.6
11	Were the methods (including statistical methods) sufficiently described to enable them to be repeated?	78.0
	**Results**	
12	Were the basic data adequately described?	90.2
13#	Does the response rate raise concerns about non-response bias?	51.2
14*	If appropriate, was information about non-responders described?	-
15	Were the results internally consistent?	73.2
16	Were the results presented for all the analyses described in the methods?	82.9
	**Discussion**	
17	Were the authors' discussion and conclusions justified by the results?	87.8
18	Were the limitations of the study discussed?	68.3
	**Other**	
19#	Were there any funding sources or conflicts of interest that may affect the authors' interpretation of the results?	65.9
20	Was ethical approval or consent of participants attained?	87.8

AXIS = Appraisal tool for Cross-Sectional Studies

Item was reverse coded (a 'No' answer to these questions meant that the criteria were met)

* Not included as an item of critical appraisal for this review

Supplementary Table S3. Results of quality assessment of each study

Author (Year)	1	2	3	4	5	6	7	8	9	10	11	12	13 †	15	16	17	18	19 †	20	Total
Choi *et al*. (2022)	Y	Y	Y	Y	Y	Y	N	Y	Y	Y	Y	Y	N	Y	Y	Y	Y	N	Y	18
Nejat *et al*. (2022)	Y	Y	Y	Y	N	Y	N	Y	Y	N	N	Y	Y	N	N	N	N	N	Y	10
Choi *et al*. (2021)	Y	Y	N	Y	N	N	N	Y	Y	Y	Y	Y	N	N	Y	Y	Y	N	Y	14
Kanimozhi *et al*. (2021)	N	Y	N	Y	Y	N	N	Y	Y	N	Y	Y	Y	N	N	N	Y	N	Y	10
Pandey *et al*. (2021)	Y	Y	N	Y	Y	Y	N	Y	Y	N	N	N	N	Y	Y	N	Y	N	Y	12
Salleh *et al*. (2021)	Y	Y	N	Y	Y	Y	N	Y	Y	Y	N	Y	Y	Y	Y	Y	Y	N	Y	15
Sarada *et al*. (2021)	Y	Y	Y	Y	Y	Y	N	Y	Y	Y	Y	N	N	Y	Y	N	Y	N	Y	16
Hamed Abdalla *et al*. (2020)	Y	Y	N	Y	Y	Y	N	Y	Y	Y	Y	Y	Y	Y	Y	Y	Y	N	Y	16
Bazrafshani *et al*. (2019)	Y	Y	N	Y	Y	Y	N	Y	Y	Y	Y	Y	Y	N	Y	Y	Y	N	Y	15
Cevik *et al*. (2019)	Y	Y	N	Y	Y	Y	N	Y	Y	Y	Y	Y	N	Y	Y	Y	Y	N	Y	17
Chotipanich *et al*. (2019)	Y	Y	N	Y	Y	Y	N	Y	Y	Y	Y	Y	Y	Y	Y	Y	Y	N	Y	16
Dehghan *et al*. (2019)	Y	Y	N	DK	Y	Y	N	Y	Y	Y	Y	Y	N	Y	Y	Y	Y	N	Y	16
Chui *et al*. (2018)	Y	Y	Y	Y	Y	Y	N	Y	Y	Y	Y	Y	N	Y	Y	Y	Y	N	Y	18
Yang *et al*. (2018)	Y	Y	N	Y	Y	Y	N	Y	Y	Y	Y	Y	Y	Y	Y	Y	Y	N	Y	16
Zulkipli *et al*. (2018)	Y	Y	N	Y	Y	Y	N	Y	Y	Y	Y	Y	N	N	Y	Y	Y	N	Y	16
Jang *et al*. (2017)	Y	Y	N	Y	Y	Y	N	Y	DK	Y	Y	Y	Y	Y	Y	Y	Y	N	Y	15
Mohd Mujar *et al*. (2017)	Y	Y	N	Y	Y	Y	N	Y	Y	Y	Y	Y	N	Y	Y	Y	Y	N	Y	16
Naja *et al*. (2017)	Y	Y	Y	Y	Y	Y	N	Y	Y	Y	Y	Y	N	Y	Y	Y	Y	N	Y	18
Oyunchimeg *et al*. (2017)	Y	Y	N	Y	Y	Y	N	Y	Y	Y	Y	Y	N	Y	Y	Y	Y	N	Y	17
Azhar *et al*. (2016)	Y	Y	N	Y	Y	Y	N	Y	N	Y	Y	Y	Y	Y	N	N	N	DK	Y	11
Dişsiz & Yilmaz (2015)	Y	Y	N	Y	Y	Y	N	Y	Y	Y	Y	Y	N	Y	Y	Y	Y	N	Y	17
Naja *et al*. (2015)	Y	Y	Y	Y	Y	Y	N	Y	Y	Y	Y	Y	N	N	Y	Y	Y	N	Y	17

Author (Year)	1	2	3	4	5	6	7	8	9	10	11	12	13 †	15	16	17	18	19 †	20	Total
Üstündağ (2015)	Y	Y	N	Y	Y	Y	N	Y	Y	Y	N	Y	N	N	Y	Y	Y	DK	Y	14
Ku & Koo (2012)	Y	Y	N	Y	Y	Y	N	Y	N	Y	Y	Y	Y	Y	Y	Y	Y	N	Y	15
McQuade *et al.* (2012)	Y	Y	N	Y	Y	Y	Y	Y	Y	Y	Y	Y	Y	Y	Y	Y	Y	N	Y	17
Puataweepong *et al.* (2012)	Y	Y	N	Y	Y	Y	N	Y	N	Y	N	Y	Y	N	Y	Y	N	DK	Y	11
Aydin Avci *et al.* (2011)	Y	Y	N	Y	Y	Y	N	Y	Y	N	Y	Y	N	Y	Y	Y	Y	N	N	15
Chow *et al.* (2010)	Y	Y	N	Y	Y	Y	N	Y	N	Y	Y	Y	N	Y	Y	Y	Y	DK	Y	15
Shih *et al.* (2009)	Y	Y	N	Y	Y	Y	N	Y	Y	N	Y	Y	Y	N	Y	Y	Y	N	Y	14
Supoken *et al.* (2009)	Y	Y	Y	Y	Y	N	N	N	Y	N	N	Y	Y	Y	Y	N	N	DK	Y	10
Tarhan *et al.* (2009)	Y	Y	N	Y	Y	Y	N	Y	N	N	Y	Y	Y	Y	Y	Y	Y	N	Y	14
Aksu *et al.* (2008)	Y	Y	N	Y	Y	Y	N	Y	N	Y	Y	N	Y	Y	Y	Y	N	DK	N	11
Ucan *et al.* (2008)	Y	Y	N	Y	Y	Y	N	Y	N	N	N	Y	Y	Y	Y	Y	N	DK	N	10
Kim *et al.* (2007a)	Y	Y	Y	Y	Y	Y	N	Y	Y	Y	Y	Y	N	Y	N	Y	Y	N	Y	17
Kim *et al.* (2007b)	Y	Y	N	Y	Y	Y	N	Y	Y	N	N	Y	N	N	N	Y	N	DK	N	10
Algier *et al.* (2005)	Y	Y	N	Y	Y	Y	N	Y	N	Y	Y	Y	Y	Y	N	Y	N	DK	Y	12
Tas *et al.* (2005)	Y	Y	N	Y	Y	N	N	Y	N	Y	Y	Y	N	Y	Y	Y	N	DK	Y	13
Yildirim *et al.* (2005)	Y	Y	N	Y	Y	Y	N	Y	Y	Y	Y	N	N	Y	Y	Y	N	N	Y	14
Gözüm *et al.* (2003)	Y	Y	N	Y	Y	Y	N	Y	N	Y	Y	Y	Y	N	Y	Y	Y	DK	Y	13
Malik & Gopalan (2003)	Y	Y	N	Y	Y	Y	N	Y	N	Y	Y	Y	Y	Y	Y	Y	N	DK	Y	13
Ceylan *et al.* (2002)	Y	Y	N	Y	Y	Y	N	Y	N	N	N	Y	N	Y	Y	Y	N	DK	N	11

* Note. Y="Yes", N="No", DK="Don't know" (this was counted as N in the 'Total' column)
† Item was reverse coded (a 'No' answer to these questions meant that the criteria were met)

Supplementary Table S4. Average score by domains of AXIS tool

Author (Year)	Quality of study design	Quality of reporting	Risk of bias	Total
Choi *et al.* (2022)	7	7	4	18
Nejat *et al.* (2022)	5	3	2	10
Choi *et al.* (2021)	5	7	2	14
Kanimozhi *et al.* (2021)	5	4	1	10
Pandey *et al.* (2021)	6	2	4	12
Salleh *et al.* (2021)	6	6	3	15
Sarada *et al.* (2021)	6	6	4	16
Hamed Abdalla *et al.* (2020)	6	7	3	16
Bazrafshani *et al.* (2019)	6	7	2	15
Cevik *et al.* (2019)	6	7	4	17
Chotipanich *et al.* (2019)	6	7	3	16
Dehghan *et al.* (2019)	6	6	4	16
Chui *et al.* (2018)	7	7	4	18
Yang *et al.* (2018)	6	7	3	16
Zulkipli *et al.* (2018)	6	7	3	16
Jang *et al.* (2017)	6	7	2	15
Mohd Mujar *et al.* (2017)	6	7	3	16
Naja *et al.* (2017)	7	7	4	18
Oyunchimeg *et al.* (2017)	6	7	4	17
Azhar *et al.* (2016)	4	5	2	11
Dişsiz & Yilmaz (2015)	6	7	4	17
Naja *et al.* (2015)	7	7	3	17
Üstündağ (2015)	5	6	3	14
Ku & Koo (2012)	6	7	2	15
McQuade *et al.* (2012)	6	7	4	17
Puataweepong *et al.* (2012)	5	5	1	11
Aydin Avci *et al.* (2011)	5	6	4	15
Chow *et al.* (2010)	5	7	3	15
Shih *et al.* (2009)	6	6	2	14
Supoken *et al.* (2009)	4	4	2	10
Tarhan *et al.* (2009)	6	6	2	14
Aksu *et al.* (2008)	4	5	2	11
Ucan *et al.* (2008)	4	4	2	10
Kim *et al.* (2007a)	7	6	4	17
Kim *et al.* (2007b)	4	3	3	10
Algier *et al.* (2005)	5	5	2	12
Tas *et al.* (2005)	5	6	2	13
Yildirim *et al.* (2005)	5	5	4	14
Gözüm *et al.* (2003)	5	7	1	13
Malik & Gopalan (2003)	5	6	2	13
Ceylan *et al.* (2002)	4	4	3	11
Mean	5.54 / 7.0	5.90/ 7.0	2.85 / 5.0	14.29 / 19.0

* Supplementary Table 2 includes nineteen questions, seven of which pertain to study design quality (2, 3, 5, 8, 17, 19, and 20). Additionally, seven questions are related to the quality of reporting (1, 4, 10, 11, 12, 16, and 18), and five questions examine the potential introduction of biases in the study (6, 7, 9, 13, and 15).

Supplementary Table S5. T&CM Modalities frequently used by Asian cancer patients during cancer treatments

Variables		N	Number of included studies	
**Nutritional Approaches**		**(n =7,912)**		
1.	Herbal medicines, pills, paste, syrup	1,480	22	[46,48,50,53,56,57,59,62,63,65-71, 74,76,77,80,81,84,85]
2.	Dietary supplement, Manufactured dietary products, Food, Nutrition therapy, Special diet, Tonics etc.	815	15	[50-52,57-62,68-72,74]
3.	Vitamins, Minerals	718	22	[46,47,50,52,54,57,59-63,65-68, 70,71,73,75-77,79]
4.	Stinging nettle (*Urtica dioica* L.)	625	8	[54,67,75-77,80,81,83]
5.	Ginger (*Zingiber officinale* Roscoe)	356	7	[46,50,53-55,66,67]
6.	Black seed, Cumin (*Habbatus sauda*)	349	6	[50,54,55,59,66,67]
7.	Animal product (tripe of goat, sheep, shark cartilage, fish oil, bird's nest, cow-urine, porcupine date, milk, quail eggs etc.)	342	11	[46,53,54,57,59,63,65-67,73,76]
8.	Mushroom	304	7	[47,53,57,59,60,63,68,79]
9.	Garlic (*Allium sativum* L.)	277	5	[48,50,55,60,67]
10.	Common mallow (*Malva sylvestris* L.) ,Dandelion	205	2	[55,67]
11.	Chamomile (*Matricaria chamomilla* L.)	203	5	[55,67,75,80,83]
12.	Fruit (Apricot, Berry, Cactus, Date, Mangosteen, Pomegranate, Raisins, Olive) or extract	195	7	[53,57,59,66,67,73,77]
13.	Water mint (*Mentha aquatic* L.)	192	1	[55]
14.	Honey, royal jelly	179	7	[46,54,57,65-67,75]
15.	White willow (*Salix alba* L.)	174	1	[55]
16.	Thyme (*Thymus vulgaris* L.)	156	3	[55,75,80]
17.	Cinnamon	149	2	[55,67]
18.	Echium Amoenum Fisch. Et Mey.	143	1	[55]
19.	Vegetable, Vegetarian food ,Green vegetable juice	125	5	[60,63,67,70,79]
20.	Soursoup (*Annona muricata* L.) (tea, pills)	111	4	[50,53,59,66]
21.	Ginseng	73	7	[47,53,57,59,60,63,79]
22.	Tulsi, holy basil (*Ocimum tenuiflorum* L.)	56	2	[46,48]
23.	Other seed , Endive seed, Flax seed, Sesame	33	5	[53,59,67,75,76]
24.	Fatty acids (Evening primrose oil, Virgin coconut oil)	31	2	[59,60]
25.	Probiotics, Kephir	30	5	[60,65-67,76]
26.	Green tea (*Camellia sinensis* L.)	30	5	[59,66,67,75,76]
27.	Grape seed	29	5	[59,67,75,76]
28.	Chlorella, Spirulina	21	3	[57,59,68]
29.	Turmeric (*Curcuma longa* L.)	13	4	[48,53,54,67]
30.	Linden tea	9	3	[67,75,83]
31.	Soy, Carob, Nut , Barley	7	3	[66,67,75]
32.	Mistletoe	7	2	[47,60]
33.	St. John's wort (*Hypericum perforatum*)	5	2	[67,75]
34.	Aloe-vera	3	2	[53,67]
35.	Rosemary (*Rosmarinus officinalis* L)	2	2	[67,75]
36.	[Small number of responses] Bark, Black bryony (*Tamus communis* L.), Black locust (*Robinia pseudoacacia* L.), Black nightshade (*Solanum nigrum* L.), Buttercup, Cannabis,marijuana, Celandine, Common chicory (*Cichorium intybus* L.), Curled parsley, Fenugreek	274	10	[48,53-55,59,60,63,67,75,83]

Variables		N	Number of included studies	
	(Trigonella foenum-graecum), Firefly wormwood, Garden dill, Gardenia, Gentian, Gingkgo, Hawthorn, Herb Vetiver (Chrysopogon zizanioides), Mint, Neem (Azadirachta indica), Phyllanthus plant (Phyllanthus niruri), Pine extract, Plantain stem (Musa paradisiaca), Rose hip, Sabah snake grass, Sage tea, Scorzenera, Soapberry tree, Spices, Thoodhuvalai (Solanum trilobatum), Watercress, Yarrow)			
37.	Others	191	6	[54,58,60,73,77,81]
**Psychological and Physical Approaches**		**(n = 2,093)**		
1.	Yoga	492	8	[46,49,51,52,57,59,70,72]
2.	Prayer	465	10	[46,50,56,57,59-61,66,68,71]
3.	Meditation, Breathing exercises	241	9	[46,50,57,61,63,65,70,77,79]
4.	Massage, Thuna, Reflexology	200	17	[46,52-54,56-61,63,65,68,70-72, 74]
5.	Exercises, Fast walking	114	2	[71,74]
6.	Acupuncture, Moxibustion	106	11	[47,52,58,60,63,68-72]
7.	Tai chi, Qigong, Reiki	96	11	[47,50,52,57-59,65,68-70,72]
8.	Music therapy	43	2	[65,71]
9.	Aromatherapy	42	4	[50,52,57,70]
10.	Spiritual therapy	41	4	[46,62,70,84]
11.	Sound, Light, Imagery therapy	40	3	[50,54,71]
12.	Enzyme therapy	40	2	[57,68]
13.	Psychotherapy	31	1	[71]
14.	Detoxification	29	3	[57,70,74]
15.	Cupping	29	5	[52,56,59,61,66]
16.	Other therapy (Bioenergy, Bloodletting therapy, Electromagnetic therapy, Healing water, Heat treatment, Humor, Hydrotherapy, Hypnotherapy, Naturopathy, Oncothermia, Ozone therapy, Spa therapy)	84	9	[47,50,52,54,60,61,65,70,71]
**Other Complementary Health Approaches**		**(n = 2,582)**		
1.	Traditional Medicine	1,005	12	[47,50,52,57-59,61,63,68,72,73, 79]
2.	Religious practices	390	8	[54,56,63,65,68,74,77,85]
3.	Ayurveda	576	6	[46,49,51,61,73,84]
4.	Homeopathy	233	8	[49-52,59,61,65,84]
5.	Home remedy	202	2	[49,51]
6.	Unani	71	1	[49]
7.	Traditional Healer	67	3	[46,57,85]
8.	Folk medicine, Leech therapy	38	2	[51,56]
**Total**		**N = 12,587**		

Supplementary Table S6. Predictors of T&CM use among Asian cancer patients

Variables	Number of studies reporting reasons
**Predictors of T&CM use**	17 [46,48,52,53,55,57-59,61-65,72,78,81,84]
Female	3 [63,78,81]
Higher household income	3 [48,78,81]
Experiencing symptoms	3 [55,60,68]
Education level	
- Higher education	2 [63,72]
- Lower education level	1 [64]
Cancer stages	
- Advanced stages	2 [81,84]
- Early stages	1 [46]
Employment status	
- Employed	1 [62]
- Unemployed	1 [48]
Previous experience with T&CM use	2 [63,72]
Ethnicity	2 [62,72]
Prior knowledge about T&CM	1 [65]
Younger age	1 [63]
Urban residence	1 [55]
Change in outlook on life after the development of cancer	1 [52]
Use of multiple chemotherapy applications	1 [81,84]
A lower degree of trust in physicians	1 [64]

Supplementary Material

Funnel plot of publication bias

To assess the presence of small-study effects, we conducted an Egger's test using the metabias command. The estimated bias coefficient was calculated as 0.43, with a standard error of 1.22, resulting in a p-value of 0.73. The test results provided weak evidence suggesting the existence of small-study effects.

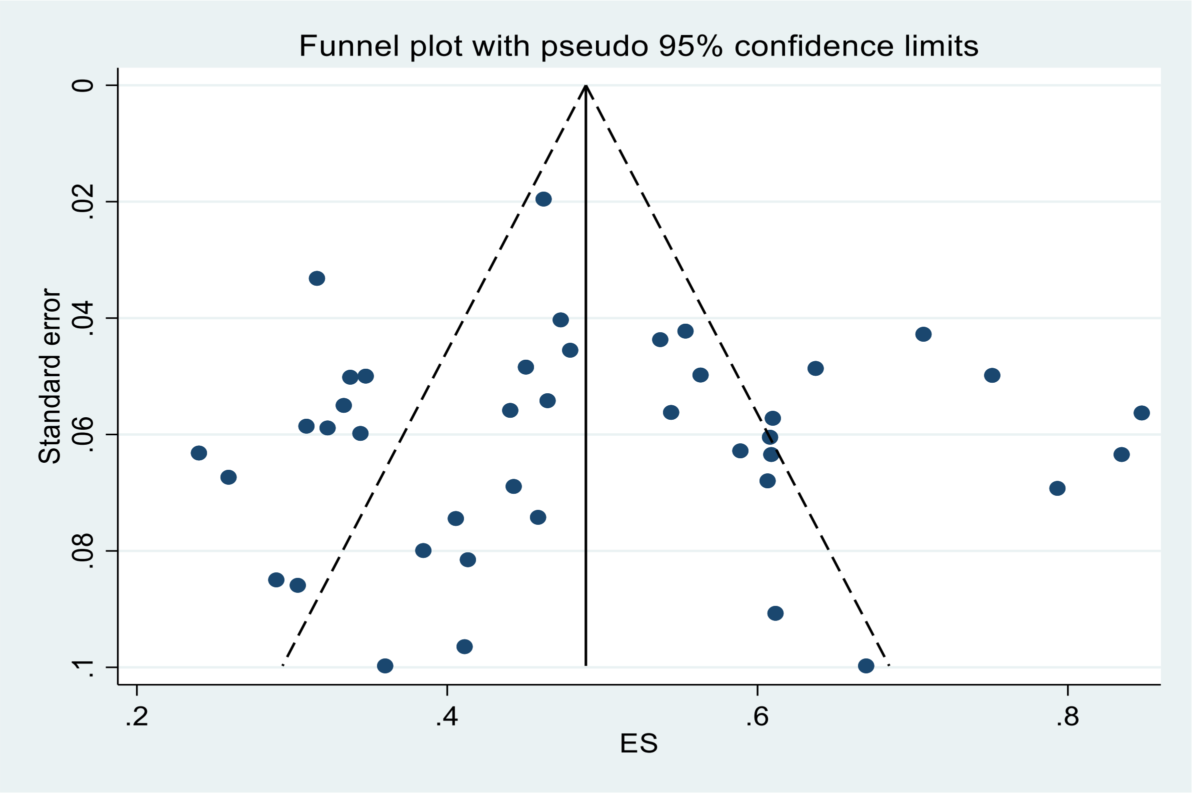

Supplementary Figure S1. Funnel plot of publication bias for 41 studies on the prevalence of T&CM use among patients with cancer

Study or Subgroup	Female Events	Female Total	Male Events	Male Total	Weight	Odds Ratio M-H, Random, 95% CI
**3.1.1 East Asia**						
Oyunchimeg et al. 2017	165	308	66	174	4.8%	1.89 [1.29, 2.76]
McQuade et al. 2012	128	148	79	100	2.6%	1.70 [0.87, 3.34]
Yang et al. 2018	141	175	161	227	4.0%	1.70 [1.06, 2.72]
Ku & Koo 2012	85	102	80	106	2.5%	1.63 [0.82, 3.22]
Kim et al. 2007a	108	185	173	338	5.0%	1.34 [0.93, 1.92]
Choi et al. 2021	30	87	60	204	3.5%	1.26 [0.74, 2.16]
Jang et al. 2017	93	150	38	66	3.1%	1.20 [0.67, 2.17]
Kim et al. 2007b	147	230	122	192	4.6%	1.02 [0.68, 1.51]
**Subtotal (95% CI)**		**1385**		**1407**	**30.0%**	**1.42 [1.20, 1.68]**
Total events	897		779			
Heterogeneity: Tau² = 0.00; Chi² = 6.46, df = 7 (P = 0.49); I² = 0%						
Test for overall effect: Z = 4.14 (P < 0.0001)						
**3.1.2 Southeast Asia**						
Salleh et al. 2021	144	227	22	46	2.8%	1.89 [1.00, 3.58]
Puataweepong et al. 2012	104	164	47	84	3.5%	1.36 [0.80, 2.33]
Chow et al. 2010	122	217	50	97	3.9%	1.21 [0.75, 1.95]
Hamed Abdalla et al. 2020	49	79	25	42	2.2%	1.11 [0.52, 2.39]
Shih et al. 2009	150	266	77	137	4.5%	1.01 [0.67, 1.53]
Chotipanich et al. 2019	77	204	115	222	4.7%	0.56 [0.38, 0.83]
**Subtotal (95% CI)**		**1157**		**628**	**21.5%**	**1.08 [0.76, 1.53]**
Total events	646		336			
Heterogeneity: Tau² = 0.12; Chi² = 14.22, df = 5 (P = 0.01); I² = 65%						
Test for overall effect: Z = 0.41 (P = 0.68)						
**3.1.3 South Asia**						
Sarada et al. 2021	70	198	26	81	3.3%	1.16 [0.67, 2.01]
Choi et al. 2022	136	458	151	450	5.8%	0.84 [0.63, 1.11]
Kanimozhi et al. 2021	39	157	18	63	2.7%	0.83 [0.43, 1.59]
**Subtotal (95% CI)**		**813**		**594**	**11.9%**	**0.89 [0.70, 1.12]**
Total events	245		195			
Heterogeneity: Tau² = 0.00; Chi² = 1.11, df = 2 (P = 0.57); I² = 0%						
Test for overall effect: Z = 1.02 (P = 0.31)						
**3.1.4 Western Asia**						
Gözüm et al. 2003	25	47	19	60	2.1%	2.45 [1.11, 5.40]
Üstündağ 2015	83	209	51	188	4.4%	1.77 [1.16, 2.71]
Ceylan et al. 2002	60	88	126	217	3.5%	1.55 [0.92, 2.61]
Bazrafshani et al. 2019	191	221	76	94	2.8%	1.51 [0.79, 2.87]
Tas et al. 2005	181	351	110	264	5.4%	1.49 [1.08, 2.06]
Aydin Avci et al. 2011	95	153	54	100	3.6%	1.40 [0.84, 2.33]
Ucan et al. 2008	165	289	145	271	5.3%	1.16 [0.83, 1.61]
Naja et al. 2017	18	44	44	106	2.4%	0.98 [0.48, 1.99]
Aksu et al. 2008	43	102	50	108	3.4%	0.85 [0.49, 1.46]
Cevik et al. 2019	42	148	51	140	3.8%	0.69 [0.42, 1.14]
**Subtotal (95% CI)**		**1652**		**1548**	**36.6%**	**1.28 [1.05, 1.58]**
Total events	903		726			
Heterogeneity: Tau² = 0.04; Chi² = 15.60, df = 9 (P = 0.08); I² = 42%						
Test for overall effect: Z = 2.39 (P = 0.02)						
**Total (95% CI)**		**5007**		**4177**	**100.0%**	**1.22 [1.07, 1.39]**
Total events	2691		2036			
Heterogeneity: Tau² = 0.06; Chi² = 52.11, df = 26 (P = 0.002); I² = 50%						
Test for overall effect: Z = 2.94 (P = 0.003)						
Test for subgroup differences: Chi² = 11.21, df = 3 (P = 0.01), I² = 73.2%						

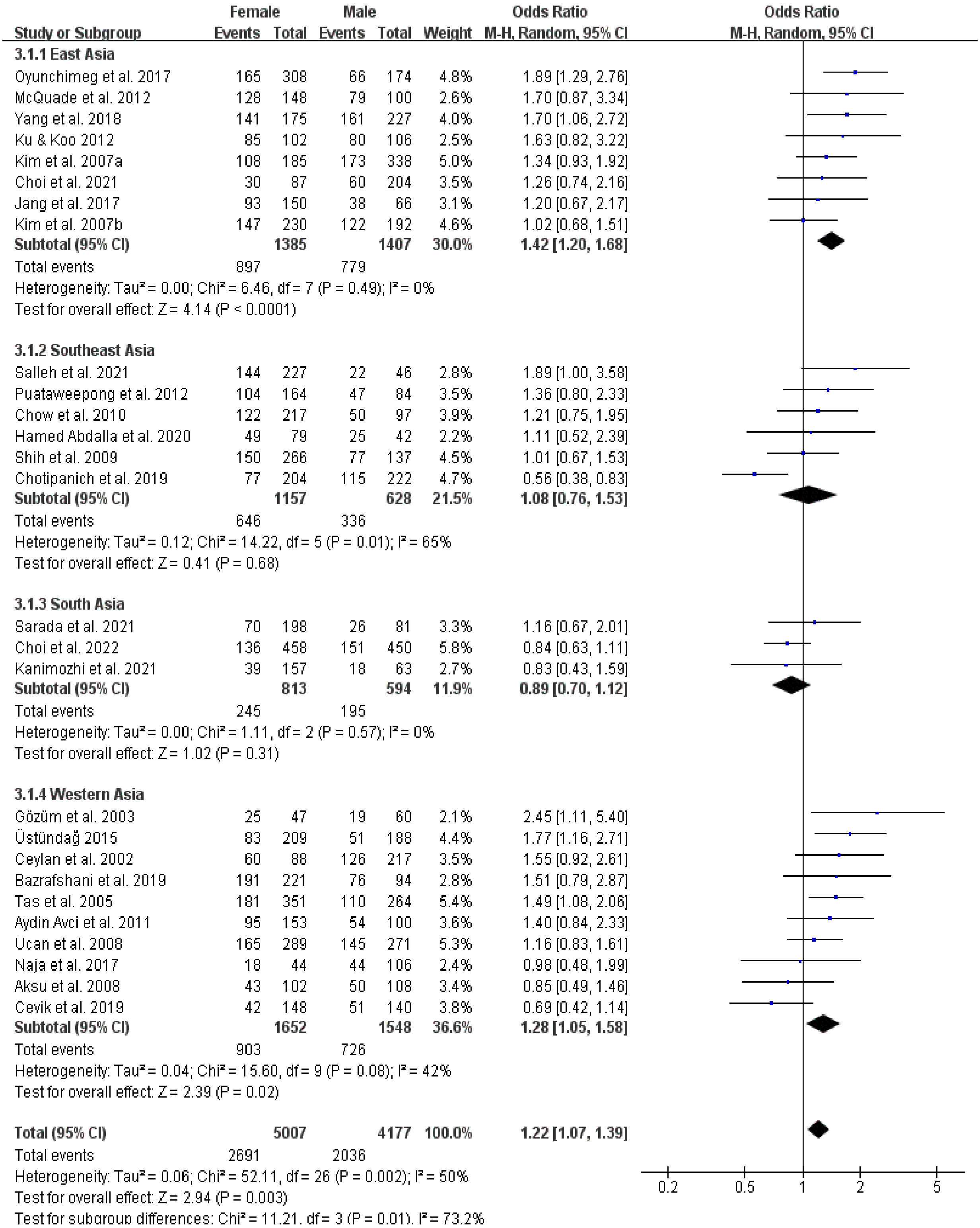

Supplementary Figure S2. Forest plot showing the odds ratio of T&CM use for females and males by location

The black diamond dots represent the individual prevalence from each study, with the horizontal lines representing the 95% CI. The green rhombus and the red dashed line represent the pooled prevalence estimate and its 95% CI.

Study or Subgroup	Female Events	Female Total	Male Events	Male Total	Weight	Odds Ratio M-H, Random, 95% CI
**3.3.1 HIGH-INCOME ECONOMIES**						
Shih et al. 2009	150	266	77	137	4.5%	1.01 [0.67, 1.53]
Kim et al. 2007b	147	230	122	192	4.6%	1.02 [0.68, 1.51]
Jang et al. 2017	93	150	38	66	3.1%	1.20 [0.67, 2.17]
Chow et al. 2010	122	217	50	97	3.9%	1.21 [0.75, 1.95]
Choi et al. 2021	30	87	60	204	3.5%	1.26 [0.74, 2.16]
Kim et al. 2007a	108	185	173	338	5.0%	1.34 [0.93, 1.92]
**Subtotal (95% CI)**		**1135**		**1034**	**24.5%**	**1.16 [0.97, 1.39]**
Total events	650		520			
Heterogeneity: Tau² = 0.00; Chi² = 1.60, df = 5 (P = 0.90); I² = 0%						
Test for overall effect: Z = 1.61 (P = 0.11)						
**3.3.2 UPPER-MIDDLE-INCOME ECONOMIES**						
Chotipanich et al. 2019	77	204	115	222	4.7%	0.56 [0.38, 0.83]
Cevik et al. 2019	42	148	51	140	3.8%	0.69 [0.42, 1.14]
Aksu et al. 2008	43	102	50	108	3.4%	0.85 [0.49, 1.46]
Naja et al. 2017	18	44	44	106	2.4%	0.98 [0.48, 1.99]
Hamed Abdalla et al. 2020	49	79	25	42	2.2%	1.11 [0.52, 2.39]
Ucan et al. 2008	165	289	145	271	5.3%	1.16 [0.83, 1.61]
Puataweepong et al. 2012	104	164	47	84	3.5%	1.36 [0.80, 2.33]
Aydin Avci et al. 2011	95	153	54	100	3.6%	1.40 [0.84, 2.33]
Tas et al. 2005	181	351	110	264	5.4%	1.49 [1.08, 2.06]
Ceylan et al. 2002	60	88	126	217	3.5%	1.55 [0.92, 2.61]
Ku & Koo 2012	85	102	80	106	2.5%	1.63 [0.82, 3.22]
Yang et al. 2018	141	175	161	227	4.0%	1.70 [1.06, 2.72]
McQuade et al. 2012	128	148	79	100	2.6%	1.70 [0.87, 3.34]
Üstündağ 2015	83	209	51	188	4.4%	1.77 [1.16, 2.71]
Salleh et al. 2021	144	227	22	46	2.8%	1.89 [1.00, 3.58]
Gözüm et al. 2003	25	47	19	60	2.1%	2.45 [1.11, 5.40]
**Subtotal (95% CI)**		**2530**		**2281**	**56.1%**	**1.27 [1.04, 1.55]**
Total events	1440		1179			
Heterogeneity: Tau² = 0.09; Chi² = 36.53, df = 15 (P = 0.001); I² = 59%						
Test for overall effect: Z = 2.34 (P = 0.02)						
**3.3.3 LOWER-MIDDLE-INCOME ECONOMIES**						
Kanimozhi et al. 2021	39	157	18	63	2.7%	0.83 [0.43, 1.59]
Choi et al. 2022	136	458	151	450	5.8%	0.84 [0.63, 1.11]
Sarada et al. 2021	70	198	26	81	3.3%	1.16 [0.67, 2.01]
Bazrafshani et al. 2019	191	221	76	94	2.8%	1.51 [0.79, 2.87]
Oyunchimeg et al. 2017	165	308	66	174	4.8%	1.89 [1.29, 2.76]
**Subtotal (95% CI)**		**1342**		**862**	**19.5%**	**1.18 [0.81, 1.72]**
Total events	601		337			
Heterogeneity: Tau² = 0.12; Chi² = 13.12, df = 4 (P = 0.01); I² = 70%						
Test for overall effect: Z = 0.85 (P = 0.39)						
**Total (95% CI)**		**5007**		**4177**	**100.0%**	**1.22 [1.07, 1.39]**
Total events	2691		2036			
Heterogeneity: Tau² = 0.06; Chi² = 52.11, df = 26 (P = 0.002); I² = 50%						
Test for overall effect: Z = 2.94 (P = 0.003)						
Test for subgroup differences: Chi² = 0.45, df = 2 (P = 0.80), I² = 0%						

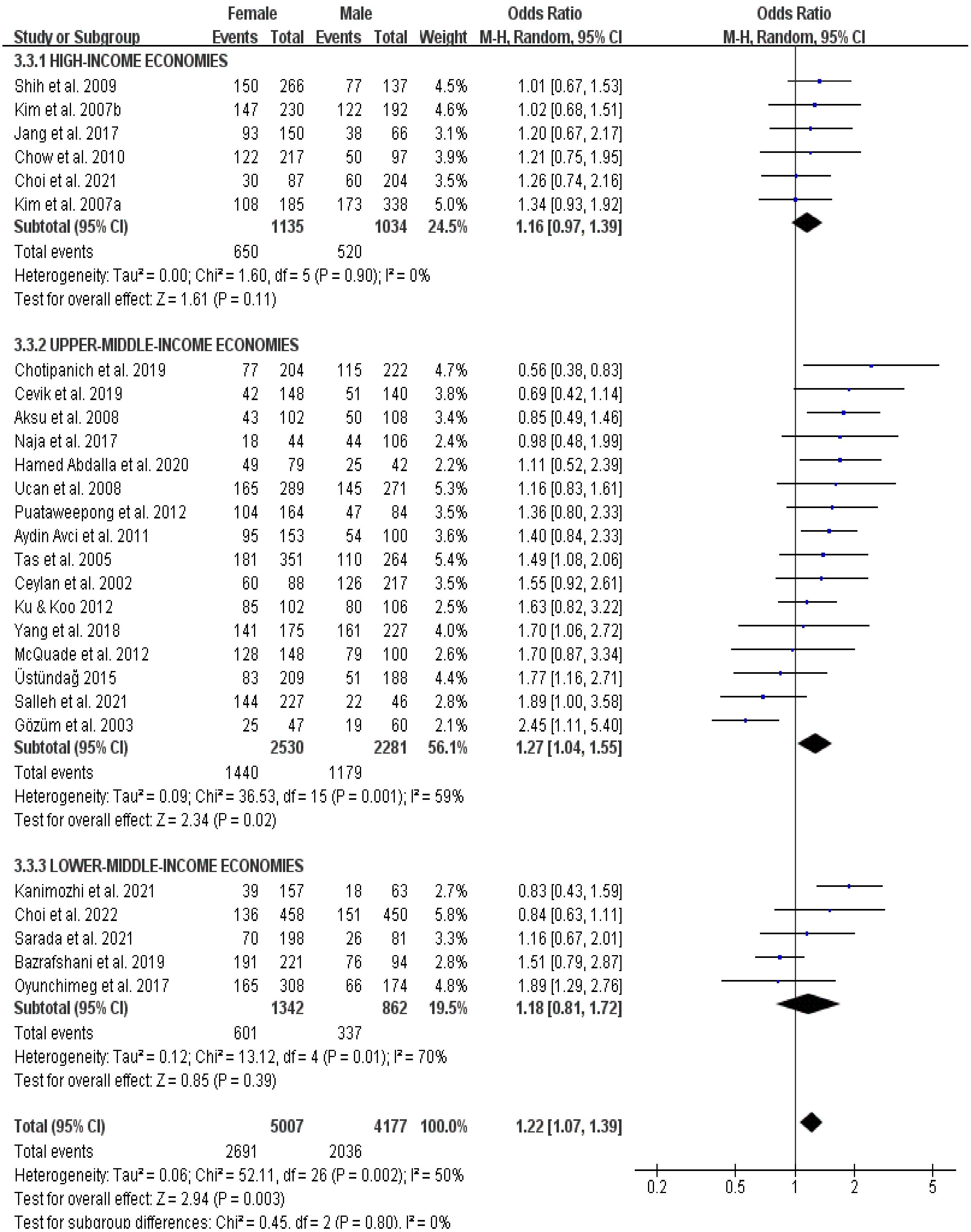

Supplementary Figure S3. Forest plot showing the odds ratio of T&CM use for females and males by the income economics

The black diamond dots represent the individual prevalence from each study, with the horizontal lines representing the 95% CI. The green rhombus and the red dashed line represent the pooled prevalence estimate and its 95% CI.

Study or Subgroup	Female Events	Female Total	Male Events	Male Total	Weight	Odds Ratio M-H, Random, 95% CI
**3.2.1 National health insurance**						
Chotipanich et al. 2019	77	204	115	222	4.7%	0.5641 [0.3833, 0.8304]
Naja et al. 2017	18	44	44	106	2.4%	0.9755 [0.4775, 1.9930]
Puataweepong et al. 2012	104	164	47	84	3.5%	1.3645 [0.7989, 2.3305]
Bazrafshani et al. 2019	191	221	76	94	2.8%	1.5079 [0.7935, 2.8653]
Oyunchimeg et al. 2017	165	308	66	174	4.8%	1.8881 [1.2920, 2.7592]
**Subtotal (95% CI)**		**941**		**680**	**18.2%**	**1.1590 [0.6923, 1.9403]**
Total events	555		348			
Heterogeneity: Tau² = 0.27; Chi² = 20.86, df = 4 (P = 0.0003); I² = 81%						
Test for overall effect: Z = 0.56 (P = 0.57)						
**3.2.2 Private health insurance**						
Cevik et al. 2019	42	148	51	140	3.8%	0.6915 [0.4210, 1.1357]
Aksu et al. 2008	43	102	50	108	3.4%	0.8454 [0.4900, 1.4587]
Shih et al. 2009	150	266	77	137	4.5%	1.0076 [0.6650, 1.5267]
Hamed Abdalla et al. 2020	49	79	25	42	2.2%	1.1107 [0.5165, 2.3881]
Ucan et al. 2008	165	289	145	271	5.3%	1.1563 [0.8284, 1.6140]
Chow et al. 2010	122	217	50	97	3.9%	1.2072 [0.7469, 1.9510]
Aydin Avci et al. 2011	95	153	54	100	3.6%	1.3953 [0.8369, 2.3263]
Tas et al. 2005	181	351	110	264	5.4%	1.4906 [1.0802, 2.0569]
Ceylan et al. 2002	60	88	126	217	3.5%	1.5476 [0.9170, 2.6119]
Üstündağ 2015	83	209	51	188	4.4%	1.7695 [1.1575, 2.7051]
Salleh et al. 2021	144	227	22	46	2.8%	1.8927 [0.9995, 3.5839]
Gözüm et al. 2003	25	47	19	60	2.1%	2.4522 [1.1129, 5.4032]
**Subtotal (95% CI)**		**2176**		**1670**	**44.7%**	**1.2721 [1.0652, 1.5193]**
Total events	1159		780			
Heterogeneity: Tau² = 0.04; Chi² = 17.70, df = 11 (P = 0.09); I² = 38%						
Test for overall effect: Z = 2.66 (P = 0.008)						
**3.2.3 Both**						
Kanimozhi et al. 2021	39	157	18	63	2.7%	0.8263 [0.4289, 1.5917]
Kim et al. 2007b	147	230	122	192	4.6%	1.0162 [0.6822, 1.5137]
Sarada et al. 2021	70	198	26	81	3.3%	1.1569 [0.6675, 2.0051]
Jang et al. 2017	93	150	38	66	3.1%	1.2022 [0.6671, 2.1667]
Choi et al. 2021	30	87	60	204	3.5%	1.2632 [0.7398, 2.1566]
Kim et al. 2007a	108	185	173	338	5.0%	1.3377 [0.9316, 1.9210]
Ku & Koo 2012	85	102	80	106	2.5%	1.6250 [0.8205, 3.2182]
Yang et al. 2018	141	175	161	227	4.0%	1.7000 [1.0610, 2.7241]
McQuade et al. 2012	128	148	79	100	2.6%	1.7013 [0.8675, 3.3364]
**Subtotal (95% CI)**		**1432**		**1377**	**31.3%**	**1.2697 [1.0717, 1.5043]**
Total events	841		757			
Heterogeneity: Tau² = 0.00; Chi² = 5.77, df = 8 (P = 0.67); I² = 0%						
Test for overall effect: Z = 2.76 (P = 0.006)						
**3.2.4 None**						
Choi et al. 2022	136	458	151	450	5.8%	0.8363 [0.6320, 1.1067]
**Subtotal (95% CI)**		**458**		**450**	**5.8%**	**0.8363 [0.6320, 1.1067]**
Total events	136		151			
Heterogeneity: Not applicable						
Test for overall effect: Z = 1.25 (P = 0.21)						
**Total (95% CI)**		**5007**		**4177**	**100.0%**	**1.2201 [1.0687, 1.3929]**
Total events	2691		2036			
Heterogeneity: Tau² = 0.06; Chi² = 52.11, df = 26 (P = 0.002); I² = 50%						
Test for overall effect: Z = 2.94 (P = 0.003)						
Test for subgroup differences: Chi² = 7.20, df = 3 (P = 0.07), I² = 58.3%						

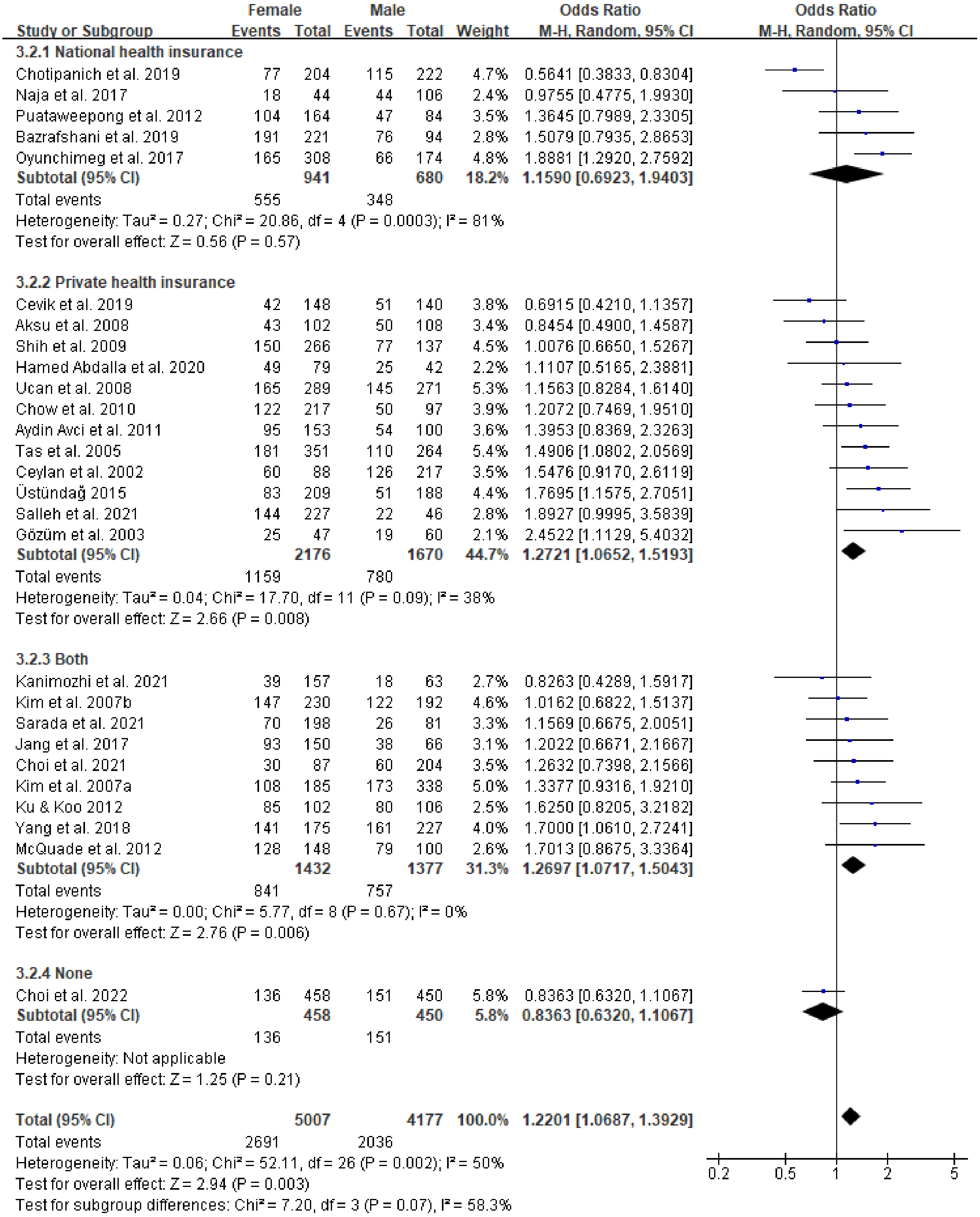

Supplementary Figure S4. Forest plot showing the odds ratio of T&CM use for females and males by the insurance

The black diamond dots represent the individual prevalence from each study, with the horizontal lines representing the 95% CI. The green rhombus and the red dashed line represent the pooled prevalence estimate and its 95% CI.

[Breast cancer]

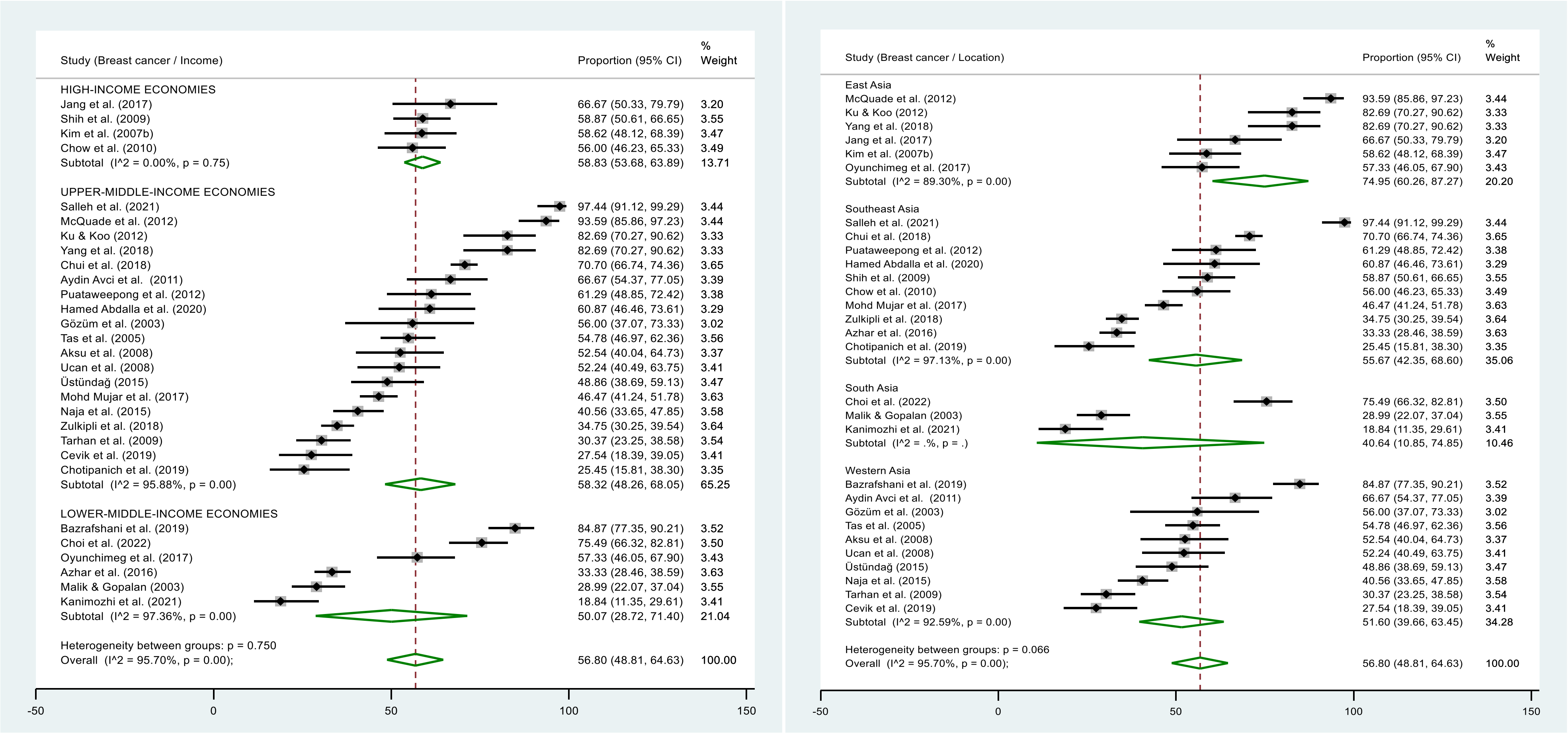

Supplementary Figure S5. Forest plot illustrating the pooled estimates of T&CM use among patients with breast cancer by income and location

The black diamond dots represent the individual prevalence from each study, with the horizontal lines representing the 95% CI. The green rhombus and the red dashed line represent the pooled prevalence estimate and its 95% CI.

[Lung cancer]

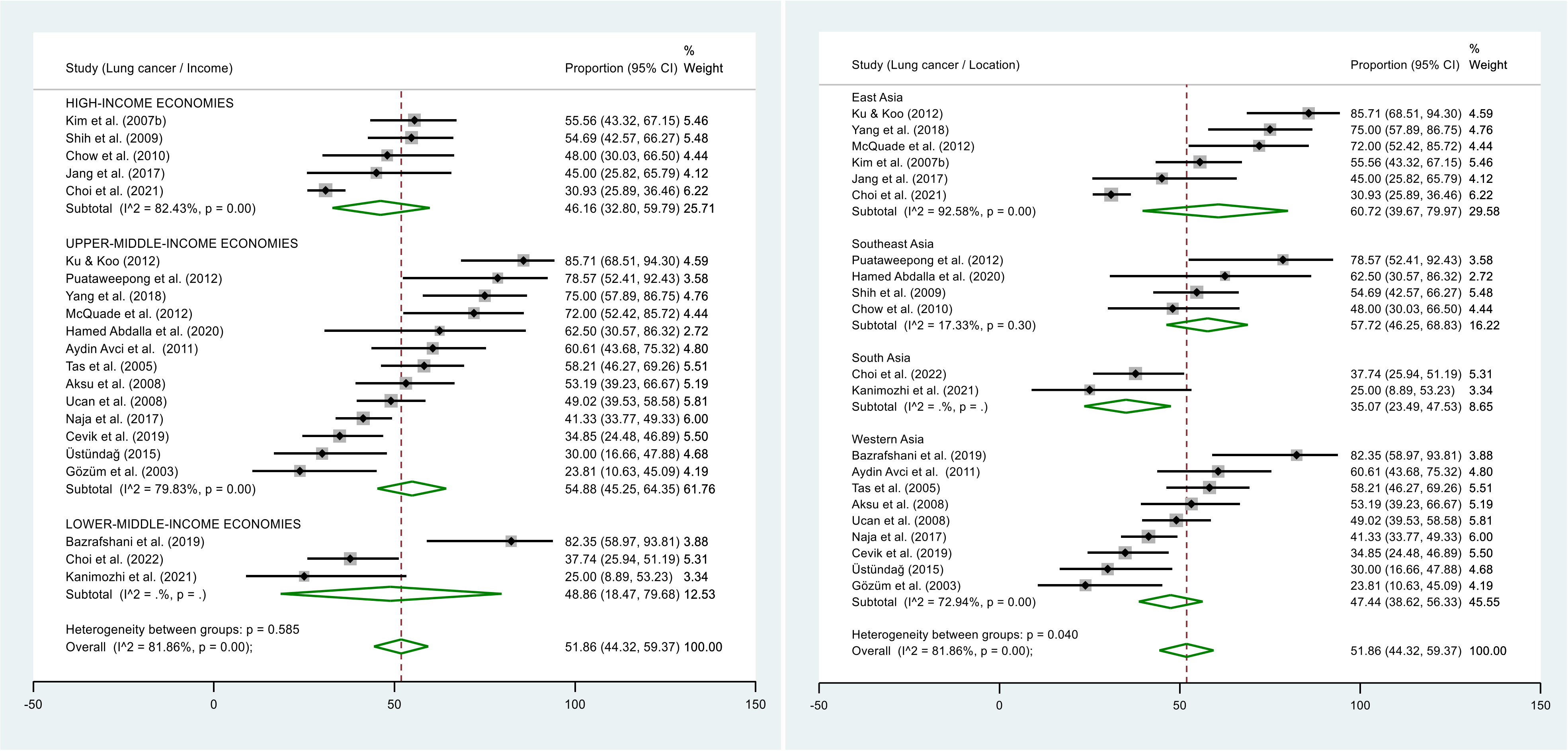

Supplementary Figure S6. Forest plot illustrating the pooled estimates of T&CM use among patients with lung cancer by income and location

The black diamond dots represent the individual prevalence from each study, with the horizontal lines representing the 95% CI. The green rhombus and the red dashed line represent the pooled prevalence estimate and its 95% CI

[Gastrointestinal cancer]

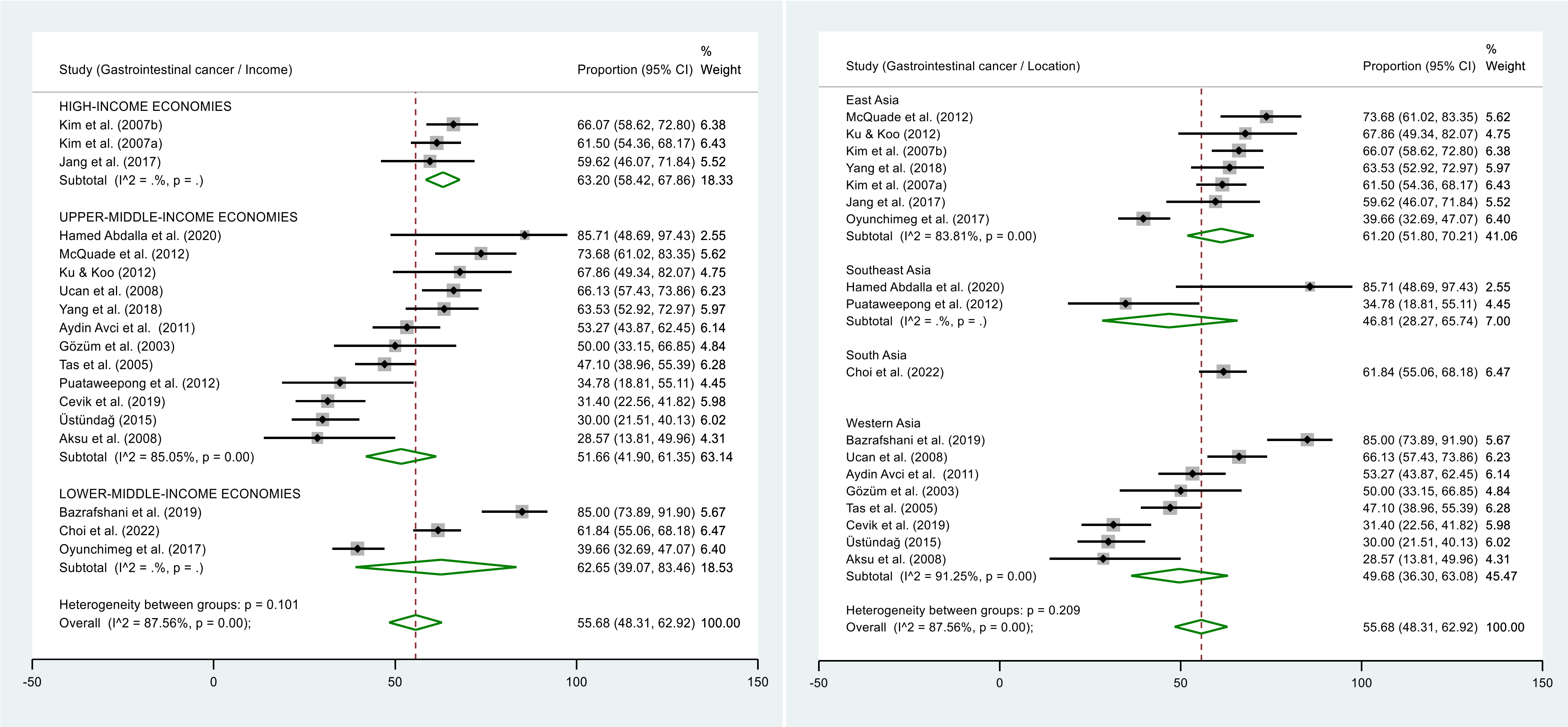

Supplementary Figure S7. Forest plot illustrating the pooled estimates of T&CM use among patients with gastrointestinal cancer by income and location

The black diamond dots represent the individual prevalence from each study, with the horizontal lines representing the 95% CI. The green rhombus and the red dashed line represent the pooled prevalence estimate and its 95% CI.

[Genitourinary cancer]

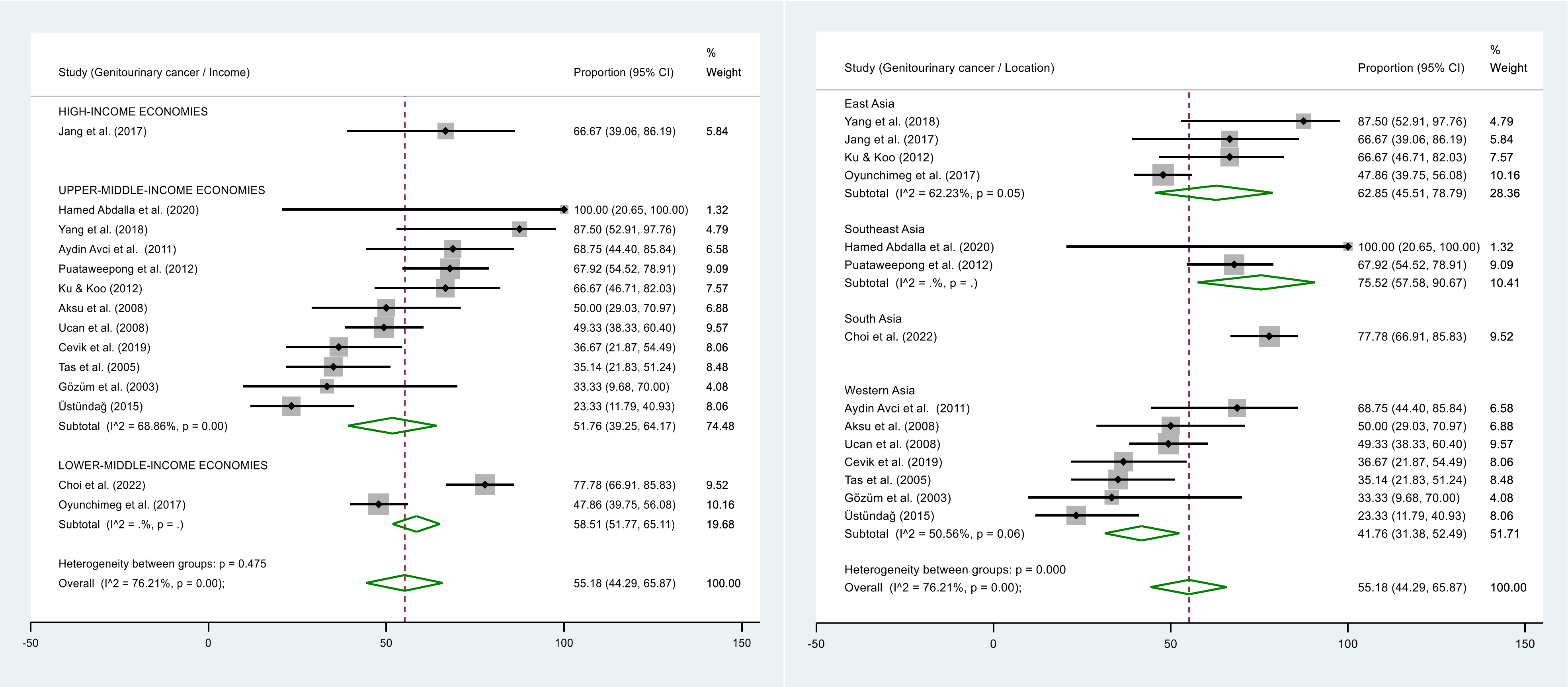

Supplementary Figure S8. Forest plot illustrating the pooled estimates of T&CM use among patients with genitourinary cancer by income and location

The black diamond dots represent the individual prevalence from each study, with the horizontal lines representing the 95% CI. The green rhombus and the red dashed line represent the pooled prevalence estimate and its 95% CI

[Head & neck cancer]

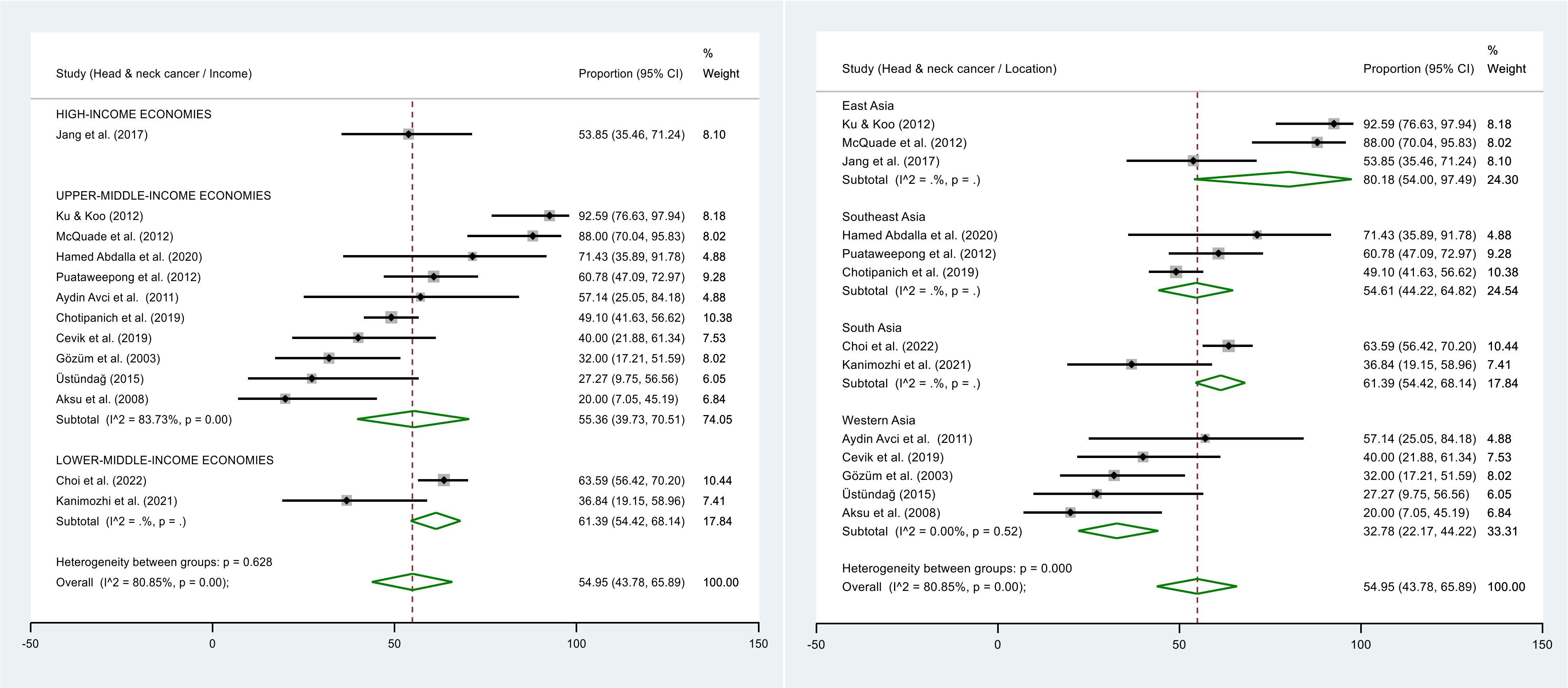

Supplementary Figure S9. Forest plot illustrating the pooled estimates of T&CM use among patients with head & neck cancer by income and location

The black diamond dots represent the individual prevalence from each study, with the horizontal lines representing the 95% CI. The green rhombus and the red dashed line represent the pooled prevalence estimate and its 95% CI

[Hematological cancer]

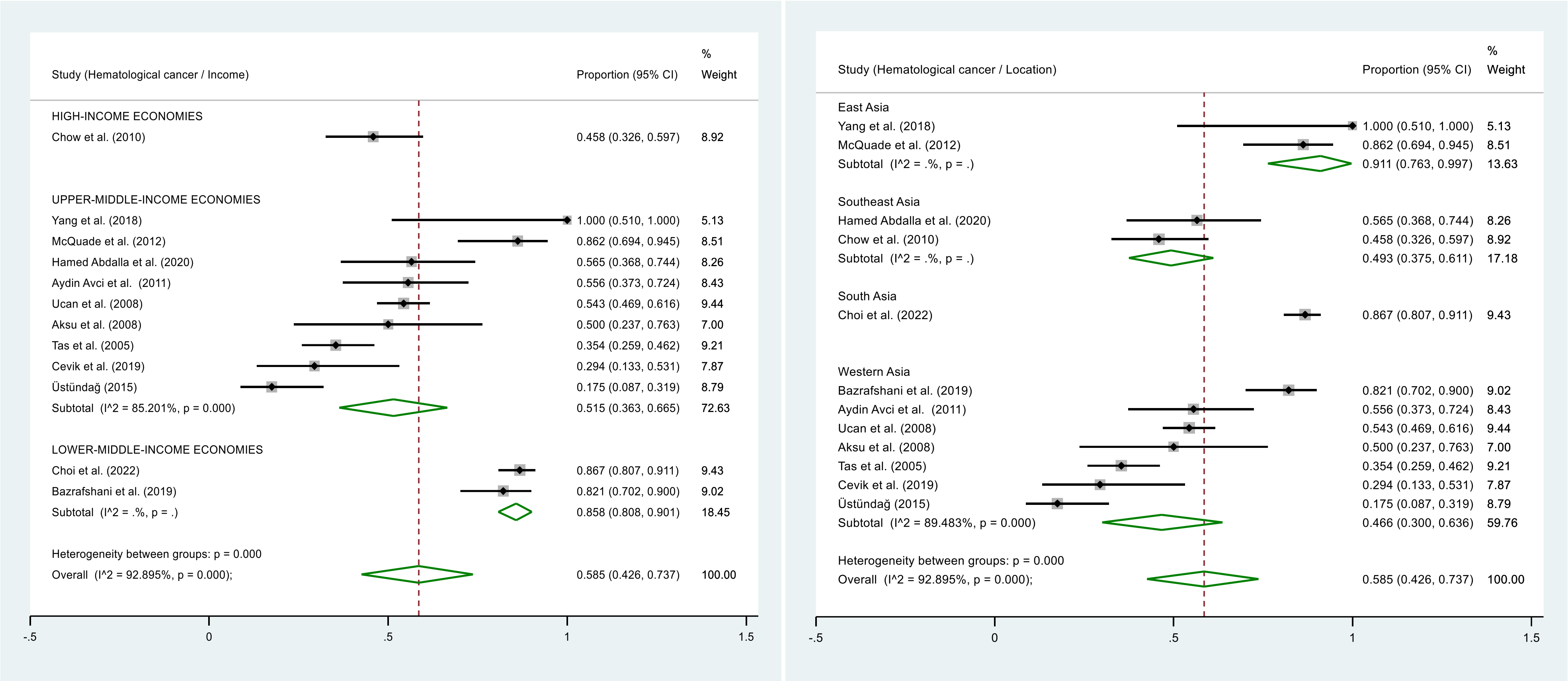

Supplementary Figure S10. Forest plot illustrating the pooled estimates of T&CM use among patients with hematological cancer by income and location

The black diamond dots represent the individual prevalence from each study, with the horizontal lines representing the 95% CI. The green rhombus and the red dashed line represent the pooled prevalence estimate and its 95% CI.

[Gynecological cancer]

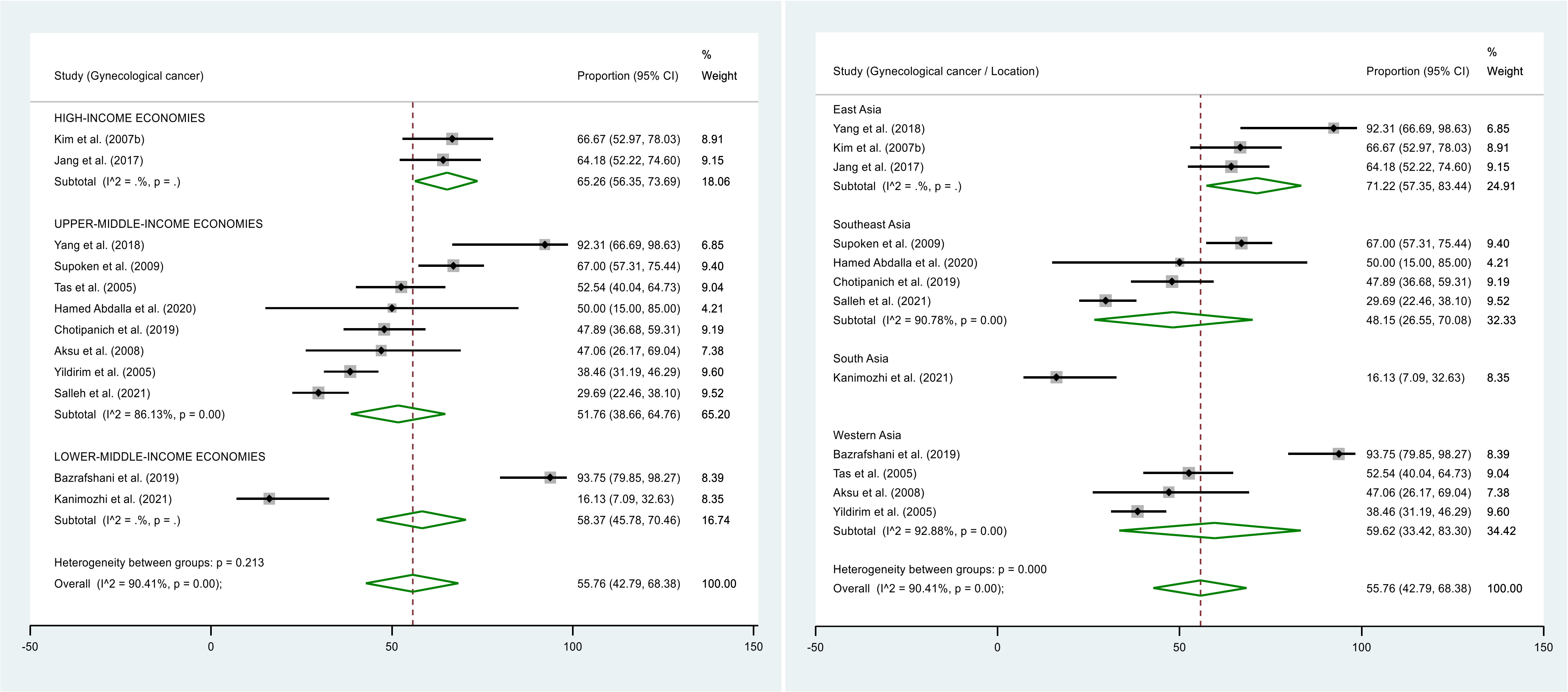

Supplementary Figure S11. Forest plot illustrating the pooled estimates of T&CM use among patients with gynecological cancer by income and location

The black diamond dots represent the individual prevalence from each study, with the horizontal lines representing the 95% CI. The green rhombus and the red dashed line represent the pooled prevalence estimate and its 95% CI.

[Colorectal cancer]

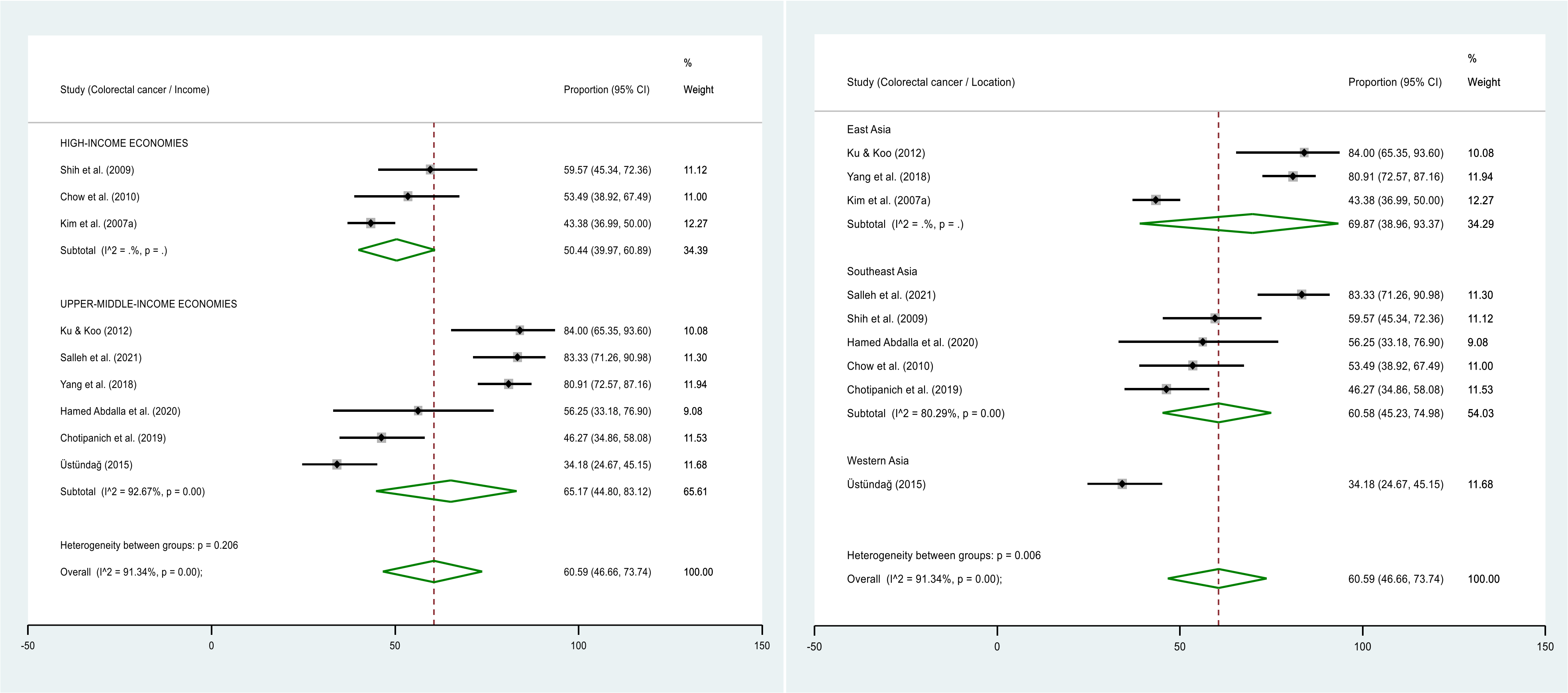

Supplementary Figure S12. Forest plot illustrating the pooled estimates of T&CM use among patients with colorectal cancer by income and location

The black diamond dots represent the individual prevalence from each study, with the horizontal lines representing the 95% CI. The green rhombus and the red dashed line represent the pooled prevalence estimate and its 95% CI.

[Hepatobiliary cancer]

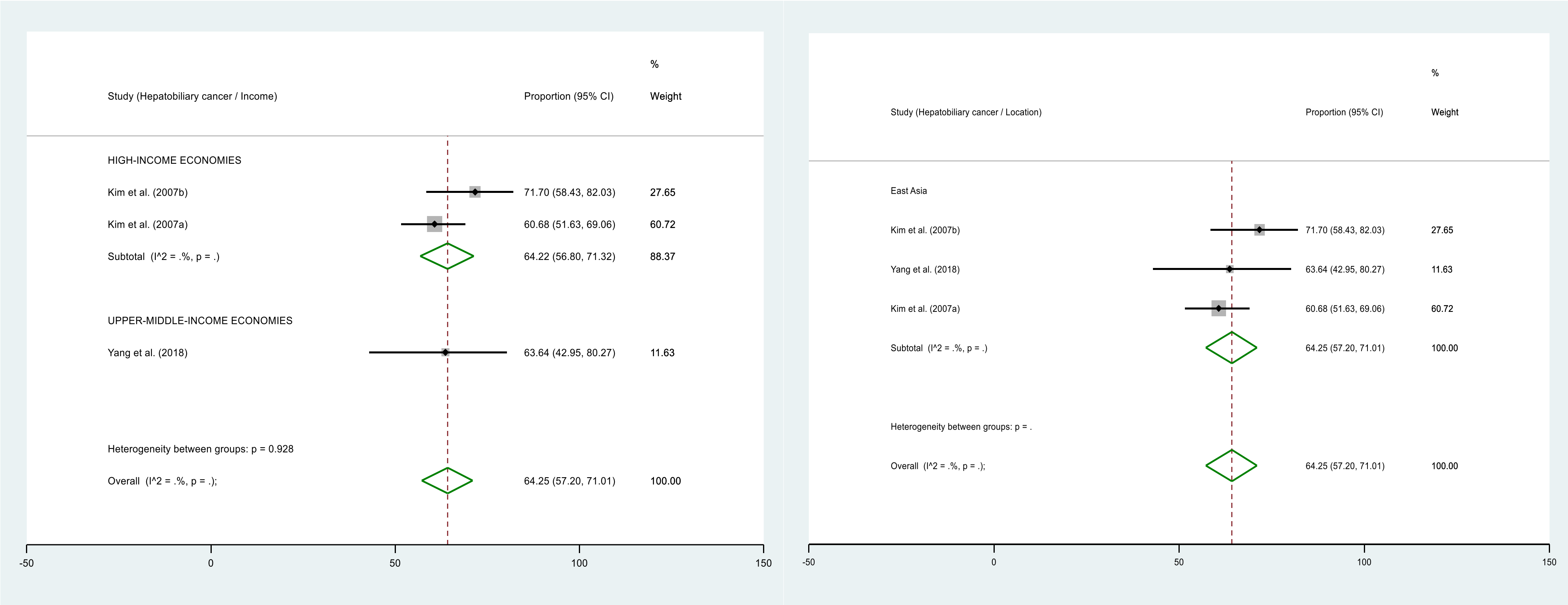

Supplementary Figure S13. Forest plot illustrating the pooled estimates of T&CM use among patients with hepatobiliary cancer by income and location

The black diamond dots represent the individual prevalence from each study, with the horizontal lines representing the 95% CI. The green rhombus and the red dashed line represent the pooled prevalence estimate and its 95% CI
